# Spatio-temporal trends in the frequency of interspecific interactions between domestic and wild ungulates from Mediterranean Spain

**DOI:** 10.1371/journal.pone.0211216

**Published:** 2019-01-25

**Authors:** Roxana Triguero-Ocaña, José A. Barasona, Francisco Carro, Ramón C. Soriguer, Joaquín Vicente, Pelayo Acevedo

**Affiliations:** 1 Instituto de Investigación en Recursos Cinegéticos, IREC (UCLM-CSIC-JCCM), Ciudad Real, Spain; 2 VISAVET Centre, Animal Health Department, Complutense University of Madrid, Madrid, Spain; 3 Estación Biológica de Doñana (CSIC), Sevilla, Spain; 4 Escuela Técnica Superior de Ingenieros Agrónomos, UCLM, Ciudad Real, Spain; Universitat Autonoma de Barcelona, SPAIN

## Abstract

Controlling infections shared by wildlife and livestock requires the understanding and quantification of interspecific interactions between the species involved. This is particularly important in extensive multi-host systems, in which controlled domestic animals interact with uncontrolled, abundant and expanding wild species, such as wild ungulates. We have, therefore, quantified the interspecific interactions between wild boar (*Sus scrofa*) and free-ranging cattle in Mediterranean Spain, along with their spatio-temporal variability. GPS-GSM-collars were used to monitor 12 cows and 14 wild boar in the Doñana National Park between 2011 and 2013. Interactions were defined as encounters between cattle and wild boar within a spatio-temporal window of 52 m and 1 hour. On average, each wild boar interacted with one cow 1.5 ± (SE) 0.5 times per day, while each cow interacted with one wild boar 1.3 ± 0.4 times per day. The frequency of interaction was significantly higher during crepuscular hours owing to the overlap of both species’ activity, and also during spring and autumn, probably owing to a higher individual aggregation around shared resources. Finally, the frequency of interaction was higher near the most significant shared resources (e.g. water points) but was lower in areas with dense vegetation. The results presented here show the usefulness of GPS monitoring as regards quantifying interactions and helping to clarify the process of pathogen transmission at the wildlife-livestock interface in Mediterranean Spain, along with the main spatio-temporal risk factors. In a changing scenario in which European populations of wild ungulates are increasing, more efficient measures with which to control interactions are required to meet the demands of farmers and managers. Our results, therefore, provide directional hypotheses that could be used to design disease control programmes.

## Introduction

Wild ungulates have, during the last few decades, begun to expand on a European level as regards both their distribution range and population size [1]. The expansion of these species causes alterations in the natural ecosystems, with notable consequences for their functioning [2]. From an epidemiological perspective, the expansion of wild ungulates increases the probability of spatial overlap with livestock production areas, and this is especially important for those which are more extensive (e.g. [3]). The interspecific interaction at the wildlife-livestock-human interface is, therefore, becoming a topic of concern as regards understanding and controlling shared infections [4], since many are zoonotic [5] and have an impact on both livestock production and the sustainable use of wildlife populations [6,7]. However, our understanding of infection dynamics and how to manage them in multi-host systems remains limited [8].

The effective management of interspecies spill-over of infectious diseases relies upon an understanding of pathogen transmission mechanisms [9]. Transmission can result from direct interactions that require close contact between a susceptible individual and an infected one, and/or from indirect interactions when a susceptible individual is infected by coming into contact with a contaminated surface, e.g., food and drinking water, or an infected vector, e.g., mosquitoes, flies, ticks, etc. (e.g. [10]). The indirect pathways are more likely to lead to pathogen transmission and disease spread across a community of host species than are the direct pathways, and the control of the indirectly transmitted pathogens is less effective when (i) the environmental persistence of the pathogen is high, and (ii) the interspecific interactions in the community of potential hosts increase [11]. There are also technological difficulties associated with detecting these interactions, mainly those involving wildlife [12–14]. The uncertainty associated with detecting and quantifying the interaction signifies that policymakers cannot be confident of the effectiveness of the husbandry and management practices designed to reduce the interspecific interaction at the wildlife-domestic interface (e.g. [15]).

Various approaches have been used to assess the relevance of the wildlife-domestic interface as regards the transmission and maintenance of shared diseases. Briefly, the interaction can be approximated by directly recording the direct and indirect interactions between individuals (e.g. [16]), and/or employing risk factor analyses to infer indirect interaction as a potential for interspecific interaction (e.g. [17]). The usefulness of a given approach depends on the epidemiological peculiarities of the system being studied and the epidemiological questions to be addressed. For instance, the role played by wildlife in disease maintenance can be indicated by information gathered in questionnaires and spatially-explicit epidemiological models (e.g. [18,19]). In the studies in which these methodologies are employed, the relevance of the interface is usually proved by the explanatory capacity of wildlife-related predictors (e.g., species presence, population abundance) in the domestic species’ disease rate and also the potential of resources being shared by domestic and wild species [20,21]. Nevertheless, the frequency with which livestock and wildlife interact can be measured using other methodological approaches, which provide a more precise rate of interactions and explicit information regarding when and where the species are interacting. Studies based on the direct observation of individuals have consequently been carried out for this purpose. However, they require a high sampling effort and are restricted to environments and species with a high detectability (e.g. [22]). In this respect, photo-trapping is a non-invasive, cost-effective approach with which to monitor interactions at target points (e.g. [10]) and it is almost independent of species/environment detectability. Finally, the frequency of interactions can be derived on the basis of specific individuals that are monitored with tracking technologies such as GPS-collars and proximity loggers, or using both devices simultaneously. Monitoring individuals, rather than target points (e.g. photo-trapping), provides a more complete description of the spatio-temporal patterns of interactions in the host community. Individual tracking systems are used to derive both direct and indirect interactions by defining spatio-temporal windows of interaction [23].

Of all the wild ungulates, the wild boar (*Sus scrofa*) is the species with the highest epidemiological importance at the wildlife-domestic interface in Europe (e.g. [24–27]). The wild boar is the most relevant wild reservoir of animal tuberculosis (TB) in the Iberian Peninsula [25,28–30]. In this respect, several studies have proved the important role played by wild boar in TB transmission and its maintenance in extensive cattle systems in the Mediterranean area of the Iberian Peninsula. Studies carried out on a regional/national scale have shown that the presence and abundance of wildlife are important risk factors as regards TB incidence in cattle [20,31–34]. In a recent study, TB prevalence in wild boar was associated with TB infection on cattle farms [19]. On a more local scale, the similarities in the habitat selected by wild boar and cattle suggested a high potential for overlapping with a seasonal variation, but the quantification of interactions between cattle and wild boar in Southern Spain has not yet been performed [21]. The only study to quantify interactions used photo-trapping [10]. This work showed the scarcity of direct interactions between domestic and wild ungulates at aggregation points, along with the relevance of water holes as regards establishing indirect interactions. These results have been reinforced through the use of proximity data loggers [34]. However, these interactions were quantified at static aggregation points, and monitoring individuals with tracking devices is, therefore, necessary in order to obtain a complete picture of the interspecific interactions among wild and domestic ungulates and their spatial and temporal patterns. We have, therefore, used GPS collars to monitor 14 wild boar and 12 cattle simultaneously (1 location·hour^-1^). This was done in order to, for the first time in this scenario, quantify interspecific interactions using spatio-temporal windows and describe their daily and seasonal variations and the main risk factors that explain these patterns on a spatial scale.

## Materials and methods

### Study area

This study was carried out in the Doñana National Park (hereafter, DNP; 37°0’ N; 6°30’ W). DNP is located in Andalusia, in the southwest of the Iberian Peninsula. The park and its surroundings have a sub-humid Mediterranean climate with an Atlantic influence: the average annual temperature is 17°C with large seasonal fluctuations, and the mean annual rainfall is 550 mm (40 years of monitoring) with high intra and interannual variability (252–1027 mm), which determines the irregularity of the river inputs (http://icts.ebd.csic.es/datos-meteorologicos). DNP is located in two lithological areas (sandy soils and silty-clay deposits) that lead to a great diversity of biotopes: marsh, beaches, mobile dunes and scrublands. All of these are the preferred habitat of large ungulates, such as red deer (*Cervus elaphus*), fallow deer (*Dama dama*) and wild boar, and also top predators, such as the Iberian lynx (*Lynx pardinus*). Finally, “la vera” is an ecotone area that is between 200 and 1500 m wide, which connects the scrublands with the marsh and in which bulrushes (*Juncus* spp.) and grasses grow. This area contains a great diversity and abundance of plant and herbivore species [20,35].

The traditional “vaca marismeña” husbandry takes place in DNP, and the number of cattle there was estimated to be 1208 in 2013. This traditional breed of cattle is endemic to the study area and is maintained in wild conditions throughout the park. These cattle are managed only once a year for routine veterinary inspection. The park is home to a moderate density of red deer (6.3 individuals/100ha, 23.54% CV), fallow deer (3.9 individuals/100ha, 25.25% CV) and wild boar (5.7 individuals/100ha, 20.63% CV) (estimations obtained with distance sampling for 2013; see [36]). From an epidemiological perspective, the incidence of TB in cattle is high within DNP (an average of 9.23% per year), and TB prevalence in wild boar (45–52%), red deer and fallow deer (14–19%) populations is also high [37,38]. DNP has, therefore, been proposed as a natural laboratory with which to describe the epidemiology of shared diseases at the wild-domestic ungulate interface.

### Sampling animals

The capture and marking of individuals were conducted between July 2011 and October 2013 following the protocol approved by the Animal Experiment Committee of Castilla-La Mancha University and by the Spanish Ethics Committee (PR-2015-03-08). The protocol was designed by specifically trained and certified scientists (B and C animal experimentation categories) according to EC Directive 86/609/EEC for animal handling and experiments.

**Fig 1:**
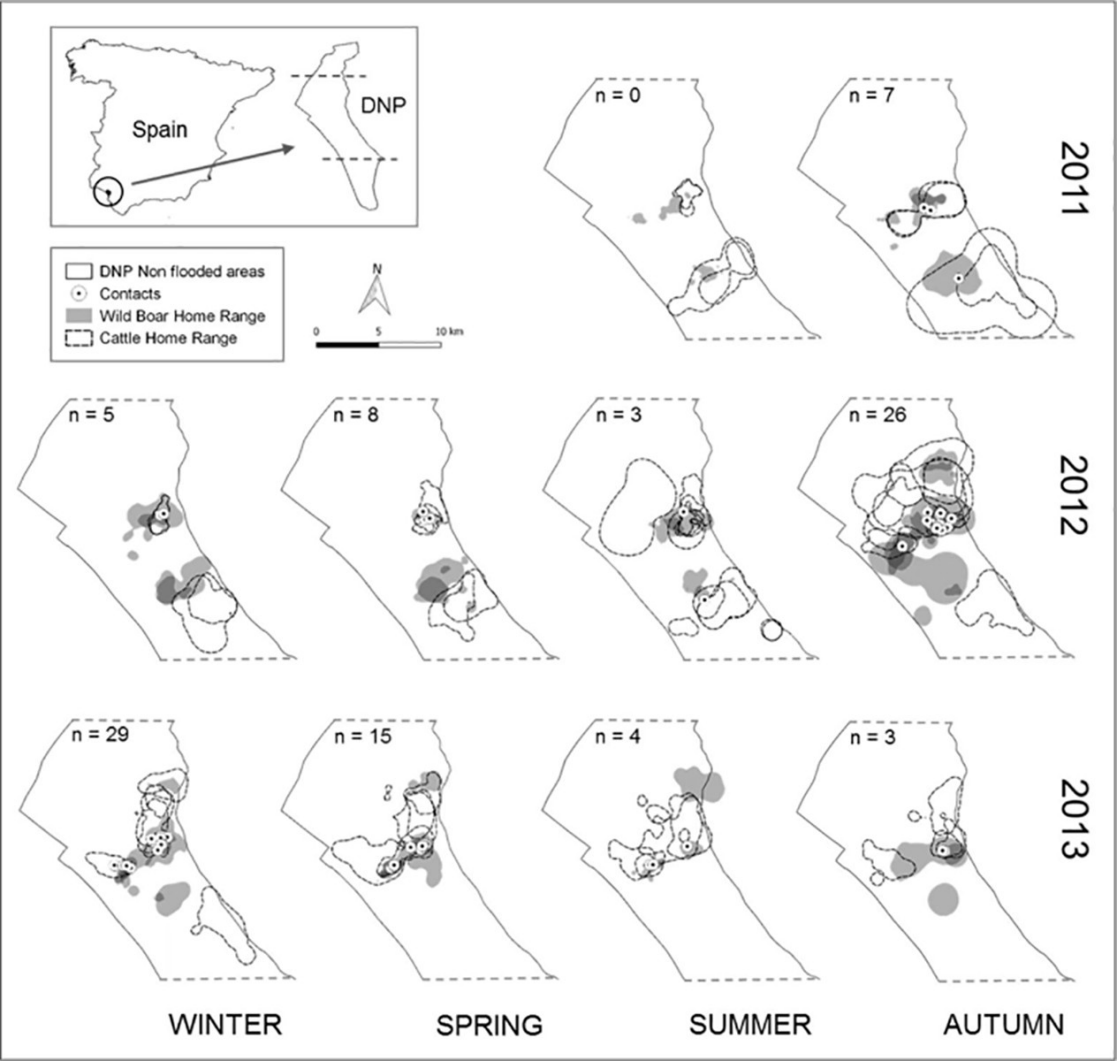
Location of the study area, Doñana National Park (DNP) Seasonal home ranges (kernel 95% UD) of the GPS-collared individuals (wild boar and cattle). The number (n) and location of interspecific interactions detected are also shown.

A total of 14 wild boar and 12 cattle (all from different social groups) were equipped with GPS-GSM radio-collars (for further details, see [21]). Individuals from different cattle herds (according to farmers’ recommendations) were collared during routine veterinary inspections in summer, using GPS-GSM devices. All the cattle were adult females. The wild boars were collared using the same GPS-GSM devices as those employed for the cattle. These wild boars were captured in different trapping areas in order to obtain individuals from different social groups. Moreover, the wild boars were captured within the cattle management areas in which various cows had previously been collared. Briefly, six padded foothold cage traps were used, each of which was activated when a wild boar stepped onto a mobile-bottomed platform in the centre of the trap, causing the simultaneous closure of the two gates on the trap. Once captured, each wild boar was anesthetised with 3 mg/kg of tiletamine-zolazepam and 0.05 mg/kg of medetomidine [39]. Ten of the 14 wild boar captured were males (9 adults and 1 sub-adult [<24 months]), and 4 were females (2 adults and 2 sub-adults). The home ranges of the targeted individuals allowed us to verify, for both species, that the animals really belonged to different social groups (see Fig 1).

Each radio-collar recorded one GPS location per hour, 24 h a day (Microsensory System, Spain). Each GPS location registered an identification for each animal (ID), date, time (solar time), geographical coordinates and location acquisition time (LAT). With regard to the last parameter, GPS locations with LAT ≥ 154 s were removed because they were considered to be anomalous relocations. In addition, GPS locations for the day of collar deployment were also discarded in order to avoid the inclusion of anomalous behaviour in the analyses associated with handling procedures. Overall, GPS fix-rate success was higher than 81% for both species, and no habitat-induced bias was found in this rate (further details can be found in [21]).

The same animals were used in a previous study carried out by our team, which aimed to explore the similarities in the habitat selection of wild boar and cattle as a means to infer a spatial index for potential interactions [21]. In the present study, GPS relocations were used to determine and quantify indirect interspecific interactions using spatio-temporal windows (see paragraph regarding “*The spatio-temporal windows”*, below) for a better understanding of the transmission process.

### Determining, quantifying and modelling the interaction rate

#### The spatio-temporal windows

When attempting to define an interaction using GPS data, it is first necessary to define two thresholds: the Euclidean distance and the critical time between two given relocations to be considered as interacting, i.e. the spatio-temporal window. These thresholds should be fitted according to the peculiarities of the target pathogen (e.g. means of transmission and/or survival rate in the environment) and/or by bearing in mind the specific characteristics/configuration of the monitoring devices. Although our study was contextualised in a typical scenario for the study of TB epidemiology (DNP), it was not focused on a given pathogen and we consequently used the specifications of the GPS devices to set the abovementioned thresholds. The spatio-temporal window has, therefore, been then defined as 52 m (26 m from the mean positional error of the GPS) and 1 h (time lag between consecutive relocations).

#### Analyses of GPS locations: Determining the interactions

The analytical rationale used to determine the interactions within the established spatio-temporal window was conducted using R software 3.3.2 [40]. For each relocation, and for both cattle and wild boar, we identified the positions of a different individual as regards the other species within the spatio-temporal window. The resulting potential interactions were subsequently characterised with the ID of each interacting individual, the Euclidean distance between relocations, the time between relocations, the date (season) and the geographical coordinates of the centroid between both interacting relocations.

#### Descriptive analysis and interaction frequency

We first employed descriptive analyses to understand individual variations in the frequency of interspecific interactions. The Shannon index (H) [41] was then estimated so as to evaluate which individuals interacted more frequently with a higher number of other collared individuals from another species.

We then estimated the interaction frequency (ifreq) in order to study the spatial and temporal patterns of indirect interactions between wild boar and cattle [42]. Briefly, for each ID and date-time that a given individual was monitored, we determined the number of interspecific interactions (#int) and the number of other collared individuals from the other species that were available for interaction (#aval). An *individual*_*j*_ was considered available to interact with an *individual*_*i*_ for a given date-time if a spatial overlap was detected in their seasonal home ranges (kernel 95% UD; [43]) and if the *individual*_*j*_ had a valid relocation at that date-time. An ifreq was subsequently estimated for each ID as: ${ifreq}_{i,x,y}=\frac{{\#int}_{i,x,y}}{{\#aval}_{i,x,y}}$; for the individual *i*, at the date *x* and at the time *y*.

Finally, we calculated the number of interspecific interactions per day for each species by multiplying the average value of specific ifreq by the number of individuals from the other species in the study area according to census values previously reported.

#### Spatio–temporal patterns

We modelled the results concerning interspecific interactions in order to describe the most relevant spatio-temporal patterns. Three temporal variables were used: (a) time was reclassified in four categories (H1: 0:00–5:59; H2: 6:00–11:59; H3: 12:00–17:59; H4: 18:00–23:59) according to the activity patterns observed in DNP for both cattle and wild boar (see S1.i File); (b) season (calendar-based seasons: Season 1 = Winter; Season 2 = Spring; Season 3 = Summer; Season 4 = Autumn), and (c) year. We also used eight environmental variables as the main spatial predictors of host abundance and aggregation [20]: surface occupied by dense shrubland (T1), low-clear shrubland (T2), herbaceous grassland (T3), woodland (T4), bare land (T5), watercourse vegetation and water body (T6), distance to water body (DW), and distance to the vera (DV). Environmental variables were measured for a 52 m buffer (according to our GPS positional error) around each relocation. Land cover data were obtained from Andalusia Environmental Information (REDIAM; Junta de Andalucía 2013). We applied a generalised mixed linear model with a negative binomial distribution and a log link function (after confirming over-dispersion when modelling with a Poisson distribution), using the ‘lme4’ package [44] in order to explain the spatio-temporal variation in ifreq, using ID as random factor. A null model that incorporated the temporal structure of the data was employed as a basis on which to follow a forward stepwise selection procedure, and Akaike´s Information Criterion (AICc) [45] was used to select the most parsimonious model. The ‘Coefplot2’ package was used to plot the results obtained from the model [46].

## Results

### Descriptive results

The information obtained from the collared individuals enabled us to record 72,906 locations, 44,437 for cattle (mean 3703 ± 2808 relocations per individual) and 28,469 for wild boar (mean 2033 ± 975 relocations per individual). One hundred interspecific interactions were identified for the spatio-temporal window established (S2 File). These interactions took place between 10 wild boar and 8 cattle. The cattle interacted more frequently with different wild boar than in the contrary case, but these differences were not significant (cattle mean H = 0.64, wild boar mean H = 0.45; Mann-Whitney U test: U = 29.5, p = 0.346). Spatial overlap analyses of the seasonal home ranges showed 24 different pairs of individuals with overlapping home ranges. In fact, on average, each cow interacted with one wild boar 1.3 ± (SE) 0.4 times per day, while each wild boar interacted with one cow 1.5 ± 0.5 times per day.

### Spatio-temporal distribution of interactions

**Fig 2:**
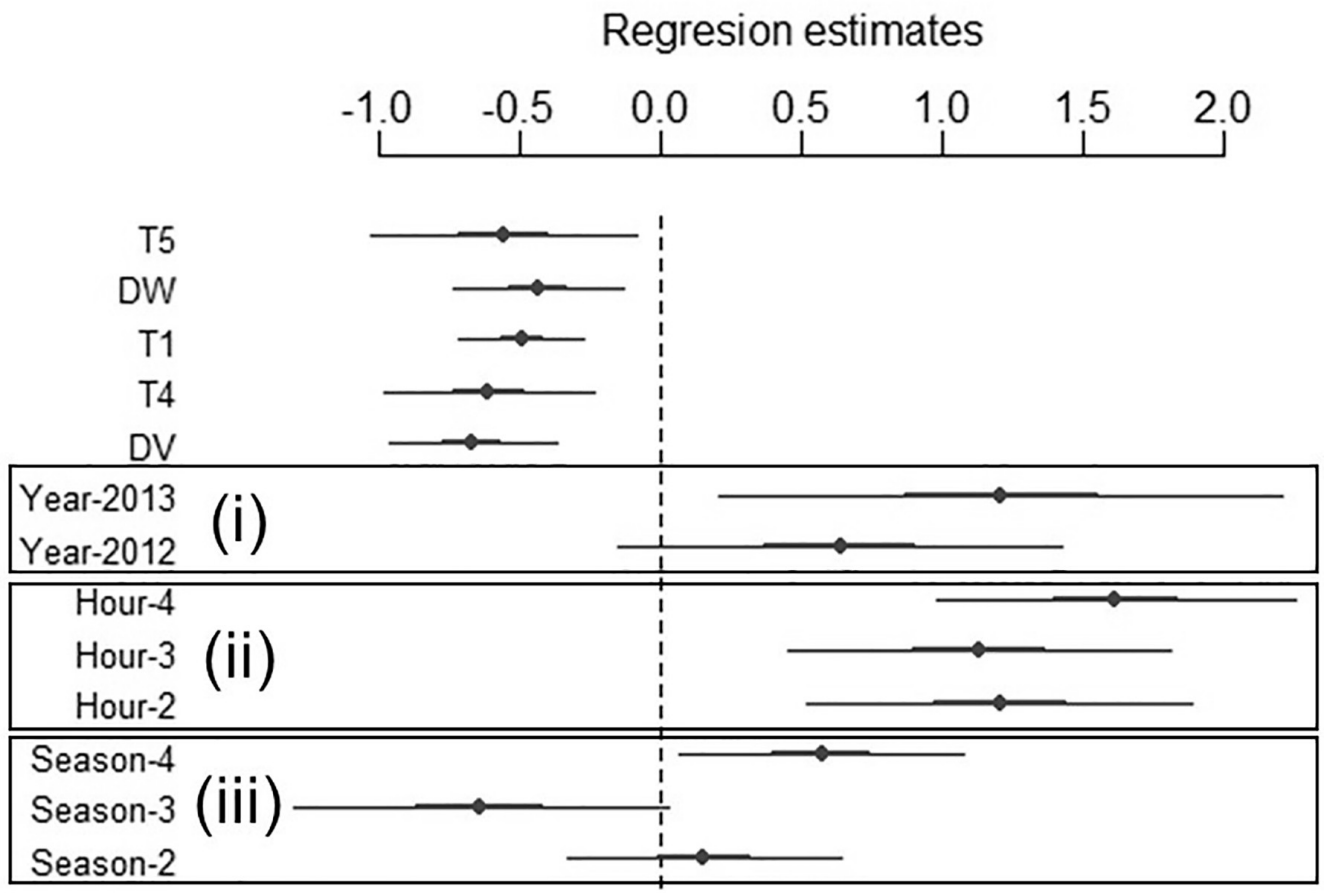
Coefficients for the predictors included in the final model performed to explain the spatio-temporal variation in the frequency of cattle–wild boar (*Sus scrofa*) interactions in Doñana National Park. The thick (inner) bars represent +/- 1 standard error, while the thin (outer) bars represent +/- 2 standard errors. T5: bare land; DW: distance to water body; T1: dense shrubland; T4: woodland; DV: distance to vera. The reference categories for the categorical predictors were: (i) Year-2011, (ii) Hour-1, and (iii) Season-1.

**Fig 3:**
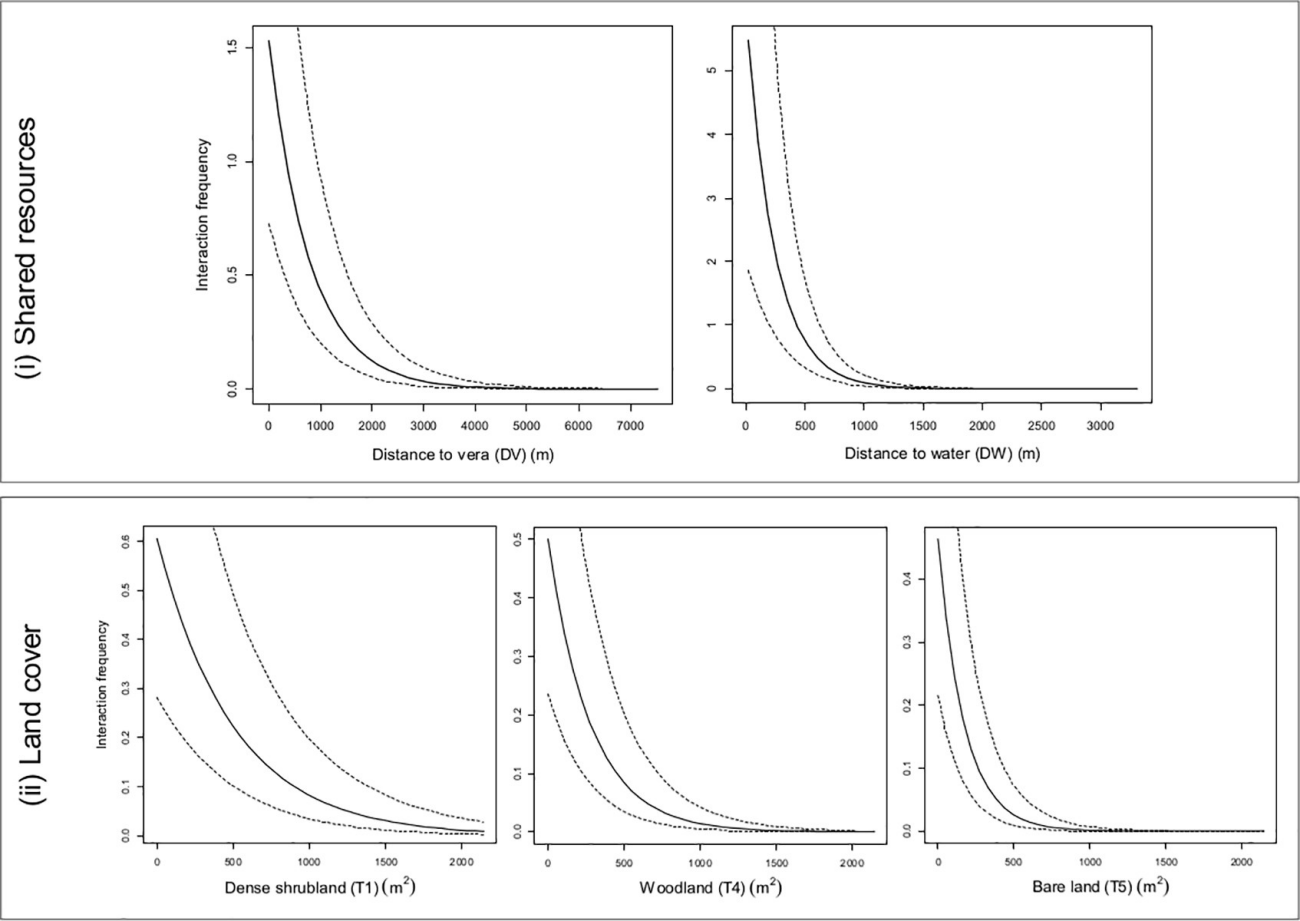
Predicted interaction frequency (ifreq) according to environmental variables included in the final statistical model: DV, DW, T1, T4 and T5. The base level of temporal predictors was: Season = 2, Hour = 2 and Year = 2012. Dashed lines represent the standard error of ifreq values. Environmental predictors appear categorised as (i) shared resources and (ii) land cover, according to the use that species make of them.

The model that was parameterised so as to identify the temporal and environmental predictors explaining interspecific interactions included the following predictors: ifreq ~ Season + Hour + Year + DV + T4 + T1 + DW + T5 (see Table 1 and Fig 2). A higher ifreq was positively associated with a close distance to the vera ecotone and water resources, and negatively associated with dense vegetation, such as woodland and shrubland, but also with areas without any vegetation (see Fig 3). Interactions occurred at crepuscular hours, mainly at the end of the day (H4), and less frequently in the middle of the night (H1). Finally, ifreq was higher in autumn and spring than in summer, and increased over the study years.

**Table 1:** Results of the best-fitting generalised mixed linear model (negative binomial distribution and log link function) to explain spatio-temporal variation in the frequency of interspecific interactions (cattle–wild boar) in Doñana National Park.

	Estimate	Std. Error	Z value	P-value
**(intercept)**	-9.98	0.59	-16.85	***
**Season2**	0.16	0.25	0.63	ns
**Season3**	-0.64	0.34	-1.89	**·**
**Season4**	0.57	0.26	2.22	*
**Hour2**	1.21	0.35	3.45	***
**Hour3**	1.14	0.35	3.27	**
**Hour4**	1.62	0.33	4.98	***
**Year2012**	0.64	0.40	1.59	ns
**Year2013**	1.21	0.51	2.37	*
**DV**	-0.67	0.15	-4.33	***
**T4**	-0.61	0.19	-3.18	**
**T1**	-0.49	0.11	-4.35	***
**DW**	-0.43	0.15	-2.79	**
**T5**	-0.55	0.24	-2.29	*

(P-values: p>0.1 “ns”; p<0.1 “·”; p<0.05 “*”; p<0.01 “**”; p<0.001 “***”)

DV: distance to vera; T4: woodland; T1: dense shrubland; DW: distance to water body; T5: bare land. Coefficients are relative to Season1, Hour1 and Year 2011, for Season, Hour and Year predictors, respectively (see Material and methods: Spatio-temporal patterns).

## Discussion

### The methodological approach

Monitoring the movement of individuals with GPS-collars is one of the most reliable approaches by which to study fine-scale interactions between species [47]. Though it enables the recording of simultaneous time-series of multiple individuals, the representativeness of the target population is not always guaranteed, since the collared individuals may not be representative of the population and the sample size might be not sufficient. In this study, we studied individuals distributed across the study area (spatial representativeness) and not outlier individuals in terms of spatial behaviour were identified.

In an epidemiological context, most studies based on GPS technology approximate the characterisation of interspecific interaction by quantifying home range overlap among individuals and/or by determining shared habitat use (e.g. [21,34,48]). However, GPS data is not often used to quantify the frequency of interspecific interactions in scientific literature. Moreover, the interesting aspect of this approach is principally that it enables pathogens with indirect transmission routes to be studied, since the precision of GPS is not usually sufficient to register direct interactions, normally closer than 3 m (but see [23,49,50]). Other techniques, such as data loggers and camera traps, also allow the detection of direct interactions [10,16,42]. However, we suggest that GPS data can complete the information obtained using these techniques by: i) providing additional information on indirect interactions to that gathered using proximity-loggers, and ii) supplying full data on the indirect interactions without the restrictions that may occur at some points monitored by photo-trapping, along with allowing the identification of individuals. The use of spatio-temporal windows with GPS data may, therefore, be an efficient alternative for a complete characterisation of the interaction frequency, mainly when working with pathogens whose indirect pathway of transmission is the most frequent, such as TB [51]. In this study we used the configuration of the monitoring devices to define the spatio-temporal windows, but they can also be defined according to the peculiarities of a target pathogen (e.g. means of transmission and/or survival rate in the environment). Technological advances in GPS-collar devices as regards attaining a higher spatial precision and longer monitoring periods will increase the usefulness of this technology in epidemiological studies.

### Cattle-wild boar interactions: Frequency and spatio-temporal patterns

The frequency of interactions has been employed in previous studies as a standardised measure of interactions among individuals per day. Proximity loggers were used by Böhm et al. [42] and Lavelle et al. [52] to attain average values of interspecific direct interaction/day of 0.03 (cattle-badger), 0.07 (badger-cattle) and 0.06 (cattle-raccoon) between livestock and wildlife. These low direct interaction rates are consistent with the avoidance behaviour of cattle and badger in UK noted by Woodroffe et al. [16]. In our specific case study, we observed that cattle and wild boars interacted an average of 1.4 times per day. Although the definition of interaction is different according to the work studied and the values of densities are unique for the whole period of study, the fact that there is a high density of ungulates in our study area (without great variations at short-term), together with a marked overlap of the activity patterns of both species (wild boar and cattle) (see S1.i File) could explain the high indirect interspecific interactions rates recorded in the present study. Our interspecific interaction rates show the great importance of this means of transmission in the study area (see also [53]). These rates could be related to the high prevalence of TB described in wild boar and the high incidence of TB in cattle, which has been shown to have a stable tendency in the last decade [20]. Specific biosecurity measures aimed to minimise interspecific interactions should, therefore, be a target for management in this area [15].

Our results have demonstrated that interspecific interactions are not evenly distributed throughout either the day or the seasons. The distribution of interspecific interactions between wild and domestic animals as regards time is modulated by the overlap in their activity periods, which are usually segregated in time [54,55]. A clear contrast between the crepuscular hours and the rest of the day was obtained in our study as regards the ifreq values, especially regarding the night hours, although the ifreq was also high in the central hours of the day (Fig 2). Previous studies have stated that wild boar in Mediterranean ecosystems is a crepuscular species [10,55]. However, the results reported here suggest that they behave in a diurnal manner, probably owing to the lack of human interference in DNP [56,57,58]. The diurnal behaviour of both cattle and wild boar, together with the wild boar’s increased activity at sunrise and sunset (S1.i File), could explain the daily distribution of the interspecific interaction frequency, which is eminently crepuscular but is not trivial during the rest of the day. This result has important implications for disease control in areas in which there is little human interference, as is the case of National Parks. Enclosing livestock during the night to prevent interactions at the wildlife-livestock interface (e.g. [22]) may not be an efficient measure in these areas, since a high proportion of boars could be active in the central hours of the day.

With regard to seasonality, spring and autumn stood out as being the most important seasons for wild boar-cattle interaction, whereas the lowest frequency of interactions was attained in the summer (Fig 2). These results coincide with those of previous studies in which a higher interspecific interaction frequency was observed during the dry seasons (summer and autumn), and near to commonly used restricted resources (e.g. [10,23]). In DNP, resources are limited at the end of summer and mainly during autumn, when wetland and water bodies dry up after months of water stress. Both livestock and wild boar consequently aggregate around water holes, which increases the probability of direct and indirect interspecific interaction. Our results are, therefore, coherent with those of previous studies carried out in DNP and in other Mediterranean areas [10,20,21,23], and reinforce the importance of the most critical season for pathogen transmission and maintenance, at least in areas with a high seasonality as regards the availability of resources.

Surprisingly, the interspecific interaction rates obtained for the summer were the lowest of the year (Fig 2). As previously stated, summer is a critical period for the potential overlap between cattle and wild boar, since the distribution of the shared resources is among the highest in the year (e.g. [21]) and high interspecific interaction rates were, therefore, expected for the summer in DNP. This, however, contrasts with the low frequencies of interactions observed in our study. The lower rates recorded here could be related to less activity–principally in the case of the wild boar–and a higher temporal segregation in the activity patterns of both interacting species (S1.ii File). Previous studies have shown a decrease in wild boar activity when there are high temperatures [59,60]. During the summer, the temperatures in DNP can reach 40°C, while the temperature drops during the night. These fluctuations in temperature could explain the lower diurnal activity recorded for wild boar and the increase in their activity during nocturnal hours. With regard to cattle, their activity drops slightly during the hottest hours in summer, but not as significantly as in the case of wild boar. These temporal patterns during summer would result in a lower probability of interaction than expected according to the potential shown in previous studies [21]. According to our results, spring is another critical season in which interspecific interactions take place. The limitation of resources in this season is probably linked to the scarcity of unflooded areas caused by the annual dynamics of the marshland [61,62]. A higher surface covered by flooded areas also aggregates the animals and consequently increases the probability of interaction. This result, therefore, highlights the importance of ecosystem dynamics in space for individuals’ aggregation beyond the traditional perception linked to the limited availability of food and water resources. Territorial restrictions, such as those derived from the marshland dynamics, are relevant especially in isolated areas that are surrounded by highly altered landscapes (“islands”), such as in DNP and other protected areas worldwide. Therefore, the particularities of this kind of ecosystems should be taken into account in epidemiological studies and in mitigation strategies for shared diseases. Measures to reduce interactions and, therefore, transmission should be designed to be particularly efficient in these risk seasons (e.g. [15]).

Our results also demonstrated spatial variation in the interaction frequency pattern according to land-cover. Livestock-wildlife interactions were low in areas with dense vegetation, such as dense shrublands and woodlands, and the role of the proximity to water points and vera was high as regards explaining the interaction pattern between domestic and wild species [10,21] (see also Fig 3). Areas with dense vegetation are used as rest points by ungulates in DNP, signifying that interactions in these areas are scarce owing to the reduced amount of activity of both livestock and wildlife. On the contrary, the water points in DNP are key resources because of their scarcity and their seasonality, and thus determine the spatial distribution and aggregation of domestic and wild ungulates [20]. Previous studies have shown the role played by water as regards explaining ungulates’ abundance [20] and their high intensity of use by both wild boar and cattle [21]. Here, we evidenced that most interspecific interactions occur around water. Water management is consequently a relevant option when managers are interested in reducing the frequency of interspecific interactions, and this has, therefore, been shown to be a workable and effective means to reduce pathogen transmission at the wildlife-livestock interface (e.g. [15]). From an epidemiological perspective, the importance of water points lies not only in their capacity for animal aggregation, but also in their optimal characteristics as regards the environmental maintenance of causal pathogens, such as foot and mouth disease [63] or TB [51,53]. In fact, previous studies have shown an association between the aggregation of animals around water points and the presence of TB-like lesions [64]. Although these kinds of measures are efficient as regards controlling the spread of diseases, they are not always feasible, since they may be too expensive for farmers. Biosecurity programmes should, therefore, be developed by bearing in mind the feasibility and social acceptance of the measures, since their objective is to be effective and functional [65].

The indirect interactions between wildlife and livestock determined in this study, along with their spatio-temporal pattern, are of great interest as regards understanding pathogen transmission in multi-host systems in the Mediterranean area, in which the climatic severity determines the distribution, abundance and aggregation of potential hosts and, therefore, their potential interactions. This knowledge is required to develop effective biosecurity programmes with which to reduce the interactions at the wildlife-livestock interface. In a scenario of clear expansion of wild ungulates in Europe, livestock farmers require these kinds of studies in order to avoid the potential risk that wild species could represent for the sustainability of an extensive livestock industry.

## Supporting information

S1 File Activity patterns (km/h) for cattle and wild boar in Doñana National Park, calculated from the locations obtained during the study period. (DOCX)

S2 File GPS data collection and number of interspecific interactions per month for each collared individual (wild boar or cattle), throughout the study period in Doñana National Park. (DOCX)

S3 File Relevant data concerning the number of recorded interspecific interaction and their date, time and coordinates. (DOCX)
