# Supplementary material for: Spatio-temporal trends in the frequency of interspecific interactions between domestic and wild ungulates from Mediterranean Spain
Source: PLoS One. 2019 Jan 25;14(1):e0211216. doi: 10.1371/journal.pone.0211216 (PMC6347242; doi:10.1371/journal.pone.0211216)
Supplement: S1 File — (DOCX) [file pone.0211216.s001.docx]

**S1. Activity patterns (km/h) for cattle (grey) and wild boar (black) in Doñana National Park calculated from the locations obtained during the study period; (i) annual activity patterns and (ii) seasonal activity patterns are shown.**
