# Supplementary material for: Spatio-temporal trends in the frequency of interspecific interactions between domestic and wild ungulates from Mediterranean Spain
Source: PLoS One. 2019 Jan 25;14(1):e0211216. doi: 10.1371/journal.pone.0211216 (PMC6347242; doi:10.1371/journal.pone.0211216)
Supplement: S2 File — (DOCX) [file pone.0211216.s002.docx]

**S2. GPS data collection and number of interspecific interactions per month for each collared individual (wild boar or cattle), throughout the study period in Doñana National Park, in addition to number of animals that interacted and Shannon index per individual.**
